# Annotations

**Published:** 1910-07-16

**Authors:** 


### ANNOTATIONS.

### The Sale of Horticultural Poisons.

SEVERAL cases have recently been before the Courts arising out of the Poisons and Pharmacy Act 1908, by which local authorities were empowered to grant licences to florists, seedsmen, etc., to sell weed-killers and insecticides containing arsenic and nicotine in districts where the existing facilities for obtaining these compounds were insufficient. These cases raise the question whether a poison licence granted to a shopkeeper covers the sale of poison by an unlicensed assistant. His Honour Judge Lumley Smith, K.C., at the City of London Court on June 28, decided that the licence which had been granted to Hobbies, Limited, did not cover their assistant who had sold a gardener's preparation containing nicotine, and he ordered the assistant, who was summoned by the Pharmaceutical Society, to pay a penalty of £5. This decision will probably be appealed against. It may be pointed out that it is illegal for the unqualified assistant of a registered chemist to sell a poison, except under the supervision of his master; but it is contended on behalf of holders of poison licences, that their licences cover their assistants.

### The Medical Report of the Port of London.

THE annual report of the Medical Officer of Health for the Port of London to December 31, 1909, is at hand, and, as usual, much curious and useful information is to be found between its blue-paper covers. During the year, 9,571 foreign vessels, carrying 14,020 passengers, and crews amounting to 62,381 men, arrived at Gravesend, out of which 2,848 vessels were medically examined. During the same period there arrived at Sheerness 337 foreign vessels, carrying 79 passengers, and crews amounting to 3,818, of which 285 vessels were inspected. The sanitary inspectors during the year made 40,356 inspections of foreign, coastwise, inland navigation vessels, and premises on shore. In 3,246 cases the quarters of the crews were condemned and cleansed. The following cases of infectious disease were reported: Cholera, 3; plague, 4; smallpox, 27; scarlet fever, 16; diphtheria, 11; enteric fever, 36; measles, 19; erysipelas, 5; continued fever, 10; relapsing fever, 1; and other diseases, including chicken-pox, 57. Only a part of these cases were treated at the Port Sanitary Hospital, of which two cases of smallpox and four of enteric died. Under the Aliens Act of 1905, 8,226 immigrants were medically examined in detail on arrival at the Port of London. In addition, 51,026 were superficially examined for diseases of an infectious nature. Of the whole number, fifteen were medically rejected, of whom two were admitted on appeal. The meat inspectors, among other work, dealt with 4,641 pig carcases imported from China, of which 8.4 per cent. were condemned as unfit for food. Of 100 pigs selected at random, portions of the diaphragm and muscular tissues of which were examined microscopically for trichina, all were reported free from disease. Some conception of the large quantities of frozen meat which are imported into this country can be had from the figures which the medical officer quotes. In addition to large quantities of fresh meat which arrive from France, Belgium, and the Netherlands, 11,067,152 carcases of sheep and lamb and 3,223,135 quarters of beef, frozen and chilled, were imported, of which numbers 67·12 and 36·35 per cent. respectively came into the Port of London. The instinct of weevils, which had escaped from a granary as the result of turning over a quantity of old wheat in the hot weather, in selecting a baker's shop in the neighbourhood as the site for their next depredations is commented upon. A number of nuisances, such as the emission of black smoke from steamers, offensive cargoes, improper sanitary arrangements, etc., are also dealt with. A number of typographical errors have crept into a report of Dr. Andrewes on the examination of meat for parasites, such as "historical" for "histological" examination, and "hydrated" for "hydatid" cyst; but, on the whole, the report is accurately and well printed in a type which it is a pleasure to read.

### Pharmacopœial Revision.

WHILE for the most part concerning the pharmacist and dispensing chemist, the second report of the Committee of Reference in Pharmacy to the Pharmacopœia Committee of the General Medical Council is of interest to the profession, by reason of one or two alterations which the committee of reference have seen fit to advise. The names of three preparations have been altered, namely, *liquor iodi fortis*, *liquor magnesii carbonatis* and *hydrargyri oleas*, respectively to *pigmentum iodi*, *liquor magnesii bicarbonatis* and *hydrargyrum oleinatum*. The following formula giving a more permanent preparation than the official one, has been substituted for it in the preparation of *linimentum hydrargyri*: *mercury ointment* 5, *ammonia solution* 4, *camphor liniment* 8; the last two being shaken together in a bottle, and the ointment then triturated with the product. To avoid the well-known and dangerous incompatibility of *liquor arsenicalis* with solutions of alkaloidal salts, especially *strychnine*, with which it is often prescribed, a new formula has been substituted which gives a solution closely resembling the official one, but with an acid reaction. *Extractum nucis vomicae* is still to be standardised to contain 5 per cent. *strychnine*, in spite of the Brussels International agreement, by which the preparation should contain 16 per cent. total alkaloids. The specific gravity of *æther* should be 0.720 instead of 0.735. On account of its being easier to make, and of its being free from objectionable colour, a plaster made with *atropine sulphate* should be substituted for *emplastrum belladonnæ*. *Extractum hydrastis liquidum* is to be standardised. The remainder of the report, in which altogether some ninety drugs and preparations are dealt with, refers to new methods of preparation and new descriptions.

